# A glycolysis-based 4-mRNA signature correlates with the prognosis and cell cycle process in patients with bladder cancer

**DOI:** 10.1186/s12935-020-01255-2

**Published:** 2020-05-20

**Authors:** Chen Zhang, Xin Gou, Weiyang He, Huaan Yang, Hubin Yin

**Affiliations:** 1 Department of Urology, The First Affiliated Hospital of Chongqing Medical University, No.1 Youyi Road, Chongqing, 400016 China; 2 Department of Gynecology and Obstetrics, The First Affiliated Hospital of Chongqing Medical University, No.1 Youyi Road, Chongqing, 400016 China; 3 Central Laboratory, The First Affiliated Hospital of Chongqing Medical University, No.1 Youyi Road, Chongqing, 400016 China; 4 Chongqing Key Laboratory of Maternal and Fetal Medicine, The First Affiliated Hospital of Chongqing Medical University, No.1 Youyi Road, Chongqing, 400016 China; 5 Department of Urology, Yubei District People’s Hospital, No. 69 Jianshe Road, Chongqing, 400016 China

**Keywords:** Glycolysis, Bladder cancer, mRNA signature, GSEA, Prognosis, PKM2, Cell cycle

## Abstract

**Background:**

Bladder cancer is one of the most prevalent malignancies worldwide. However, traditional indicators have limited predictive effects on the clinical outcomes of bladder cancer. The aim of this study was to develop and validate a glycolysis-related gene signature for predicting the prognosis of patients with bladder cancer that have limited therapeutic options.

**Methods:**

mRNA expression profiling was obtained from patients with bladder cancer from The Cancer Genome Atlas (TCGA) database. Gene set enrichment analysis (GSEA) was conducted to identify glycolytic gene sets that were significantly different between bladder cancer tissues and paired normal tissues. A prognosis-related gene signature was constructed by univariate and multivariate Cox analysis. Kaplan–Meier curves and time-dependent receiver operating characteristic (ROC) curves were utilized to evaluate the signature. A nomogram combined with the gene signature and clinical parameters was constructed. Correlations between glycolysis-related gene signature and molecular characterization as well as cancer subtypes were analyzed. RT-qPCR was applied to analyze gene expression. Functional experiments were performed to determine the role of PKM2 in the proliferation of bladder cancer cells.

**Results:**

Using a Cox proportional regression model, we established that a 4-mRNA signature (NUP205, NUPL2, PFKFB1 and PKM) was significantly associated with prognosis in bladder cancer patients. Based on the signature, patients were split into high and low risk groups, with different prognostic outcomes. The gene signature was an independent prognostic indicator for overall survival. The ability of the 4-mRNA signature to make an accurate prognosis was tested in two other validation datasets. GSEA was performed to explore the 4-mRNA related canonical pathways and biological processes, such as the cell cycle, hypoxia, p53 pathway, and PI3K/AKT/mTOR pathway. A heatmap showing the correlation between risk score and cell cycle signature was generated. RT-qPCR revealed the genes that were differentially expressed between normal and cancer tissues. Experiments showed that PKM2 plays essential roles in cell proliferation and the cell cycle.

**Conclusion:**

The established 4‑mRNA signature may act as a promising model for generating accurate prognoses for patients with bladder cancer, but the specific biological mechanism needs further verification.

## Background

Bladder cancer is the 10th most common cancer in the world, with an estimated 80,470 new cancer cases and 17,670 deaths in the United States in 2019; thus it is a great threat to human health [1]. Bladder cancer is a heterogeneous disease with two major clinical subtypes: non-muscle‐invasive bladder cancer (NMIBC) and muscle‐invasive bladder cancer (MIBC). Over 70% of bladder cancer patients are diagnosed with NMIBC, which has a high rate of recurrence but a low mortality [2]. However, up to 20–25% of patients are identified at first diagnosis as having MIBC. MIBC is the cause of the majority of deaths from bladder cancer, and it has unsatisfactory long-term survival and a high risk of distant metastasis [3]. The adverse outcomes of MIBC may be attributed to an insufficient understanding of its molecular characteristics and biological mechanisms as they relate to tumorigenesis and development. Therefore, it is of vital importance to identify reliable prognostic biomarkers that can predict clinical outcomes and inform decisions about observation, diagnosis, surgery, pharmacological intervention and conservative treatments.

Bladder cancer not only is an invasive disease but also is an energy metabolic disease. Reprogrammed energy metabolism is a characteristic of cancer [4]. Cancer cells exhibit increased glycolysis, which is characterized by the excessive conversion of glucose to lactic acid regardless of oxygen availability; this process is known as the “Warburg effect” [5]. It has become the most important metabolic marker in almost all cancer cells. Increased glycolysis provides energy to cancer cells and heightens the potential for the production of glycolytic intermediates [6]. Glycolysis is an attractive early target for cancer treatment, as the activated “Warburg effect” is positively correlated with tumor malignancy, implying that glycolysis may play important roles in predicting the clinical outcome of cancer patients [7]. Therefore, it is that the relationship between glycolysis and tumors be clarified, which would contribute to a better understanding of the mechanism of tumorigenesis and the development of bladder cancer.

In this study, using the TCGA database, we developed a 4-mRNA signature based on glycolysis-related gene sets to predict the survival of patients with bladder cancer. The predictive performance of the glycolysis-related gene signature was validated using GEO datasets. Additionally, a nomogram based on the 4-mRNA signature and clinical factors was constructed to assess clinical significance. GSEA was utilized to identify underlying biological processes and molecular mechanisms implicated in tumorigenesis and the development of bladder cancer, such as the cell cycle, hypoxia, p53 and PI3K/AKT. Finally, we determined the expression of the four genes and the effect of PKM2 on bladder cancer cells and found that PKM2 played an important role in the regulation of cell growth.

## Materials and methods

### Data collection

RNA expression data and clinical information were downloaded from the Cancer Genome Atlas (TCGA) data portal (https://portal.gdc.cancer.gov/). The analyzed specimens were recorded with complete RNA-seq data and detailed information about overall survival (OS) status and corresponding follow-up time. A total of 405 bladder cancer patients and 19 normal bladder tissues were included for the subsequent study. This research follows the access rules and publication guidelines of TCGA.

The mRNA expression profile matrix files of GSE31684 and GSE32548 were downloaded from the GEO database (https://www.ncbi.nlm.nih.gov/geo/) and were analyzed as validation cohorts. Ninety-three samples with OS, disease-specific survival (DSS) and recurrence-free survival (RFS) from GSE31684 and 128 samples with DSS from GSE32548 were chosen for external validation [8, 9]. Detailed information is shown in Table 1.

**Table 1:** Summary of baseline clinical pathological parameters of patients with bladder cancer in the three datasets

Characteristic	TCGA	GSE31684	GSE32548
Age (years)			
≤ 65	160	29	44
> 65	245	64	87
Gender			
Male	299	68	100
Female	106	25	31
Grade			
Low	22	6	55
High	382	87	75
T stage			
Ta	1	5	40
T1	3	10	51
T2	117	17	38
T3	193	42	0
T4	58	19	0
N stage			
N0	235	49	N/A
N1–3	128	28	N/A
AJCC stage			
I–II	130	29	N/A
III–IV	273	64	N/A
LVI			
Negative	128	N/A	N/A
Positive	149	N/A	N/A
Survival status			
Alive	227	28	105
Deceased	178	65	25
Mean follow-up time (month)	26.6	47.5	50.4

N/A, not applicable

In this study, detailed information on the molecular subtypes of bladder cancer, p53-like signature score, epithelial-mesenchymal transition (EMT) signature score, cell cycle signature score, carcinoma-in situ (CIS) signature score and TP53 mutation was obtained from previous research [10], as shown in Additional file 1.

### Collection of clinical samples

Fifteen cases of cancer specimens and paired adjacent non-cancerous tissues were collected from patients diagnosed with primary bladder cancer in the Department of Urology of the First Affiliated Hospital of Chongqing Medical University. Ethics approval required was obtained from the local hospital ethic committees and a written consent was signed by each patient before sample collection. Information on ethics approval is shown in Additional file 2. The clinical information of the patients is provided in Additional file 3. The specimens were frozen and stored at − 80 °C until used for RNA isolation.

### Gene set enrichment analysis (GSEA)

To identify glycolysis-related gene sets in 19 bladder cancer tissues and paired normal tissues from TCGA cohort, analysis was performed using GSEA software 3.0 from the Broad Institute [11]. The Hallmark gene sets (h.all.v6.1.symbols.gmt), BioCarta gene sets (c2.cp.biocarta.v7.0.symbols.gmt), KEGG gene sets (c2.cp.kegg.v7.0.symbols.gmt), PID gene sets (c2.cp.pid.v7.0.symbols.gmt) and Reactome gene sets (c2.cp.reactome.v7.0.symbols.gmt) were downloaded from the Molecular Signatures Database (https://www.gsea-msigdb.org/gsea/msigdb/genesets.jsp). For each analysis, gene set permutations were performed 1000 times to obtain a normalized enrichment score (NES), which was used for sorting pathways enriched in each phenotype. Finally, the gene set was determined for subsequent analysis when normalized P < 0.05, false discovery rate (FDR) < 0.1 and |NES| > 1.6, and thirty-nine genes from the REACTOME_GLYCILYSIS gene set were identified as core genes.

### Construction and validation of the gene signature

The mRNA data in the TCGA cohort were used as the training set. Univariate Cox regression analysis was conducted to identify the OS-related core genes, and genes with P < 0.1 were utilized for the subsequent multivariate Cox regression analysis. Following the multivariate analysis, we established a glycolysis-based 4-mRNA signature for generating prognoses, and the risk score for each patient was calculated as follow: risk score = (β1 × expression of gene1) + (β2 × expression of gene2) + (β3 × expression of gene3) + (β4 × expression of gene4). All patients were split into either high-risk or low-risk groups according to the median risk score.

Based on the 4-mRNA signature and classification of the median risk score, the mRNA expression profile matrix files of GSE31684 and GSE32548 were analyzed as validation sets.

### Establishment and assessment of the nomogram

The nomogram combining the 4-mRNA signature with clinicopathologic characteristics was plotted to predict the 3- and 5-year survival of patients with bladder cancer via the ‘rms’ package of R software (version 3.5.1). Calibration plots and time-dependent ROC curves were generated to evaluate the performance of the nomogram. In the calibration graph, nomogram predicted clinical outcomes are presented on the x-axis and y-axis, respectively; the 45‑degree dotted line indicates the ideal prediction.

### Protein network construction

GeneMANIA (http://www.genemania.org/), a website based database and tool for predicting interactions and functions of genes and gene sets on the basis of multiple networks [12], was used to develop a 4-mRNA-involved network and to screen hub genes in the regulatory network; the determined weight reflects the data source relevance for predicting the function of interest.

### RNA extraction and reverse transcriptase quantitative polymerase chain reaction (RT‑qPCR)

Total RNA was extracted from bladder cancer tissues and cell lines using TRIzol reagent (Invitrogen; Thermo Fisher Scientific, Inc.) according to the manufacturer’s instructions. Complementary DNA (cDNA) was synthesized using 1 µg of total RNA and a PrimeScript RT reagent kit (Takara) according to the manufacturer’s instructions. RT‑qPCR was performed using SYBR Green assays (Takara), which were executed by ABI 7500 Real‑Time PCR system (Applied Biosystems). The relative mRNA expression was calculated using the 2^−ΔCq^ method [13], and β‑actin was used as a loading control. Primer sequences (Invitrogen; Thermo Fisher Scientific, Inc.) are listed in Table 2.

**Table 2:** The information of four mRNAs associated with overall survival in patients with bladder cancer

Gene	Ensemble ID	Location	β (cox)	HR	P
NUP205	ENSG00000155561	chr7:135,242,662-135,333,505	0.236	1.266	0.049
NUPL2	ENSG00000136243	chr7:23,221,446-23,240,630	-0.286	0.751	0.101
PFKFB1	ENSG00000158571	chrX:54,959,394-55,024,967	-0.132	0.876	0.124
PKM	ENSG00000067225	chr15:72,491,370-72,524,164	0.154	1.166	0.110

### Cell culture and small interfering RNA transfection

Human bladder cancer cells (T24 and 5637) were purchased from the American Type Culture Collection (ATCC). The 5637 and T24 cells were cultured in RPMI-1640 medium (Corning) with 10% fetal bovine serum (FBS, Gbico, USA), 100 mg/ml penicillin and 100 mg/ml streptomycin at 37 °C in 5% CO_2_.

Small interfering RNA (siRNA) was purchased from GenePharma Biological Technology (Shanghai, China). Transfections were performed using Lipofectamine 2000 (Invitrogen; Thermo Fisher Scientific, Inc.) according to the manufacturer’s protocol. Cells were transfected with PKM2 siRNAs (siRNA‑1: sense, GCCAUAAUCGUCCUCACCA; siRNA‑2: sense, CCAUAAUCGUCCUCACCAA) or negative control siRNA (sense, CUUACGCUGAGUACUUCGA) at a concentration of 50 nM for 6 h. After 48 h, the treated cells were collected for subsequent experiments.

### Western blot

Western blot assays were performed according to the standard protocol [14]. Total protein was extracted using RIPA lysis buffer (Beyotime, Haimen, China) containing 1% PMSF (Beyotime). The protein concentration was calculated by a bicinchoninic acid kit (Beyotime). Protein was subjected to 12% SDS–polyacrylamide gel electrophoresis and then was transferred to PVDF membranes (Merck Millipore, Darmstadt, Germany). The membranes were blocked with 5% skim milk, incubated with primary anti-PKM2 (Proteintech, 15822-1-AP), anti-PKM1 (Proteintech, 15821-1-AP) and anti–β-actin (Proteintech, 20536-1-AP) overnight; then they were incubated with a goat anti-rabbit secondary antibody labeled with horseradish peroxidase (Cell Signaling Technology, Inc., Danvers, MA, CST #7074) and were detected by an enhanced chemiluminescence detection system (Biorad, USA).

### Cell proliferation assay

T24 and 5637 cells were seeded into 96-well plates at a density of 5 × 10^3^ cells per well. Cell growth was determined using the Cell Counting Kit-8 (CCK8) assay, wherein 10 μl of CCK8 solution was added per well. After incubation for 1 h, absorbance at 450 nm was measured using a microplate reader (Infinite 200 PRO, TECAN, Männedorf, Switzerland). All experiments were performed in triplicate. In addition, cell proliferation was also analyzed using a Cell-Light EdU Apollo 567 in vitro kit (C10310-1; Guangzhou RiboBio Co., Ltd.) according to the manufacturer’s instructions. Images were visualized and captured under a fluorescence microscope (Olympus Corporation).

### Cell cycle analysis

T24 and 5637 cells were fixed in 70% ice-cold ethanol for 24 h at 4 °C. Then, the cells were centrifuged and washed with sterile PBS, incubated with 100 µl of RNase A (0.1 mg/ml) for 30 min at 37 °C, stained with 2 µl of propidium iodide (PI, Sigma–Aldrich, St. Louis, MO, USA) and incubated for 30 min in the dark. Finally, cell cycle distribution was analyzed using a FACSCalibur flow cytometer (BD Biosciences, Franklin Lakes, NJ, US).

### Statistics

Statistical analyses were conducted by IBM SPSS 22.0 software (SPSS, Chicago, IL) and GraphPad Prism 5.0 software (San Diego, CA). The correlations between clinicopathological parameters and risk score were assessed using a Chi square test. Univariate and multivariate Cox proportional hazard regression analyses were performed to evaluate the prognostic significance of each factor. The prognostic outcome was assessed by Kaplan–Meier curve and log‑rank test. Two tailed Student’s t tests were utilized to compare differences between two groups. Comparisons among multiple groups were performed using one-way analysis of variance (ANOVA), followed by the Newman-Keuls post hoc test. Correlations among each signature were analyzed by the Spearman rank correlation test. Genetic alterations of the 4 glycolysis-related genes in bladder cancer were inquired from cBioPortal website (http://www.cbioportal.org/). Quantitative data are shown as the mean ± standard deviation (SD). The heatmaps, multiple GSEA, forest plot, ROC curves and calibration plots were drawn using Rstudio (version 3.5.1). P < 0.05 was considered statistically significant.

## Results

### Identification of glycolysis-related genes via GSEA

**Fig. 1:**
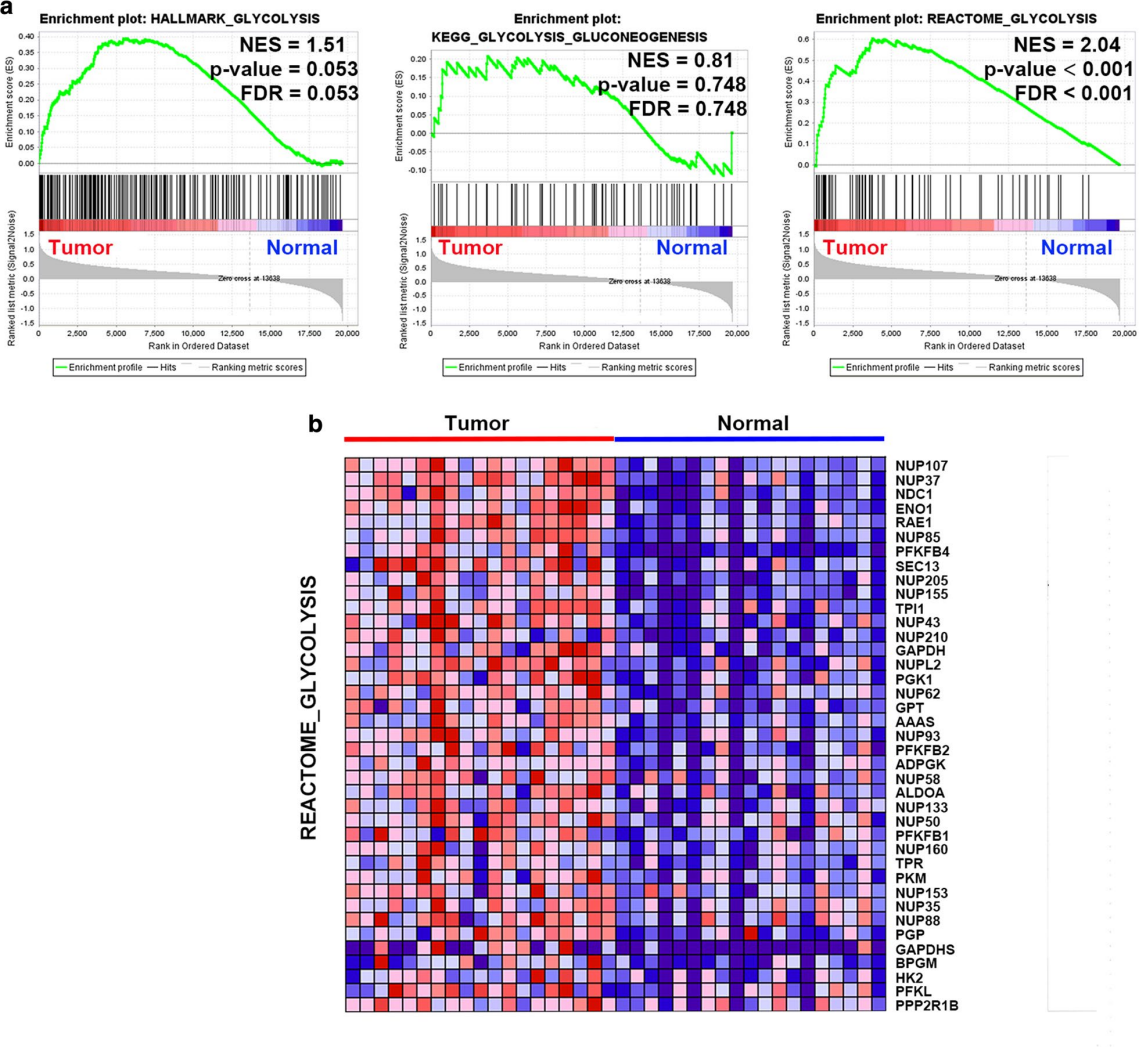
GSEA of glycolysis-related gene sets. **a** Enrichment plots of three glycolysis-related gene sets between bladder cancer and paired normal tissues identified by GSEA. **b** Heatmap of 39 core genes from the REACTOME_GLYCOLYSIS gene set between bladder cancer and paired normal tissues

GSEA was performed to explore whether three glycolysis-related gene sets were significantly different between bladder cancer samples and paired adjacent normal samples. The results showed that only the REACTOME_GLYCOLYSIS gene set was significantly enriched with cancer samples (NES = 2.04, nominal P < 0.001, FDR < 0.001) (Fig. 1a). Thirty-nine core genes in the REACTOME_GLYCOLYSIS gene set were screened (CORE ENRICHMENT: YES); that is, the genes whose expression was up-regulated in cancer samples were used in further analysis (Fig. 1b).

### Identification of glycolysis-related genes associated with survival of bladder cancer patients

Core genes were analyzed by univariate Cox regression for preliminary screening, of which ten genes were associated with OS (P < 0.1). We next used the multivariate Cox regression method to examine the association between the expression profiles of ten genes expression and the survival of patients. Subsequently, 4 mRNAs, NUP205, NUPL2, PFKFB1 and PKM, were verified as independent indicators of poor prognosis. Thees filtered mRNAs were classified into a risk role (NUP205 and PKM) with hazard ratio (HR) > 1 and a protective role (NUPL2 and PFKFB1) with HR < 1 (Table 3). A prognostic model based on a signature of the 4 mRNAs was developed to assess the survival risk of each patient as follows: risk score = (0.2360 × expression of NUP205) + (− 0.2861 × expression of NUPL2) + (− 0.1323 × expression of PFKFB1 + (0.1539 × expression of PKM).

**Table 3:** Primer sequence

Gene	Forward (5′–3′)	Reverse (5′–3′)
PKM	ATGTCGAAGCCCCAAGTGAA	TGGGTGGTGAATCAATGTCCA
NUP205	GTACTGGGATGGAAAGCGATG	GCTCTGGACTGAGTTCTAGGG
PFKFB1	GGCCAGTATCGACGAGAGG	CAAAAACCGCAACATGACCTTC
NUPL2	GCTTTGGATTGTCTGAGAACCC	CAAGCCTCAATTCCTCTGGTG
β-actin	CATGTACGTTGCTATCCAGGC	CTCCTTAATGTCACGCACGAT

**Fig. 2:**
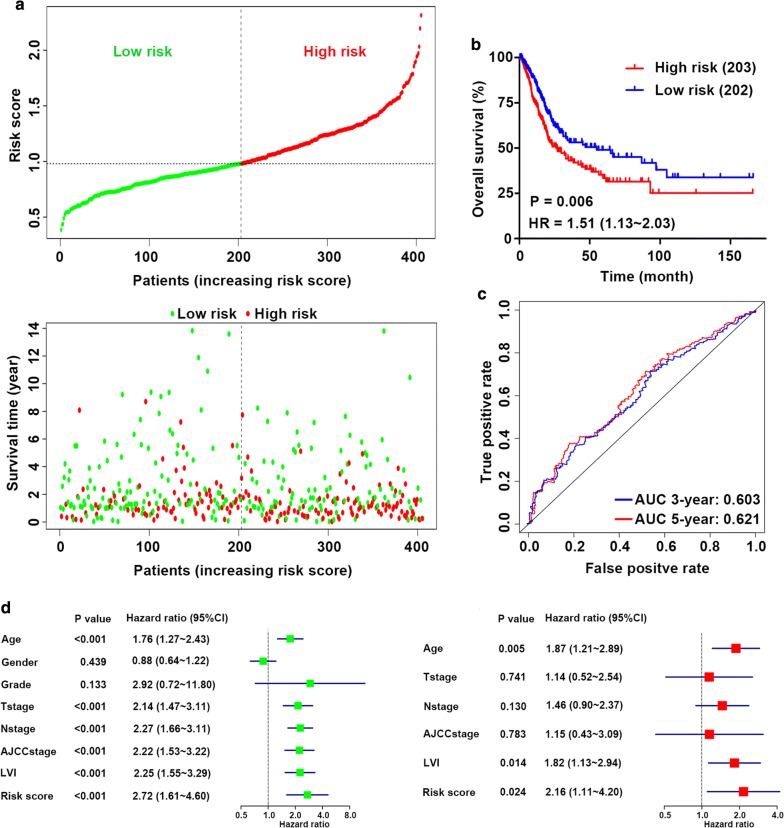
Risk score based on the 4-mRNA signature predicts OS in patients with bladder cancer. **a** The distribution of the 4-mRNA risk score and survival status for each patient. **b** Kaplan–Meier curve of OS in high- and low-risk groups. **c** Time-dependent ROC curves of the 4-mRNA signature for prediction of 3- and 5-year OS. **d** Univariable and multivariable analyses for the risk score and each clinical feature

Using the 4-mRNA signature, we classified patients with bladder cancer in TCGA cohort into high and low risk groups based on the median value. The distribution of risk score and survival status for each patient is exhibited in Fig. 2a, suggesting that patients in the high-risk group had a higher mortality than those in the low-risk group. Similarly, a Kaplan–Meier survival curve and a log-rank test showed that patients with a high-risk score had a poorer OS than those with a low-risk score (Fig. 2b). The time‑dependent ROC curve showed that the areas under the curve (AUC) at 3‑ and 5‑year were 0.603 and 0.621, respectively (Fig. 2c), indicating appropriate sensitivity and specificity of the 4-mRNA signature in predicting survival for patients with bladder cancer.

### Risk score based on the 4-mRNA signature acts as an independent prognostic indicator

To test the predictive ability of the glycolysis-related risk score for OS in TCGA cohort, we compared the prognostic value of the 4-mRNA signature with several clinicopathological features via univariate and multivariate analyses. Clinical factors included age, gender, grade, T stage, N stage, American Joint Committee on Cancer (AJCC) stage and lymphovascular invasion (LVI). The results of univariate analysis indicated that age [HR = 1.76, 95% confidence interval (CI) 1.27–2.43, P < 0.001], T stage (HR = 2.14, 95% CI 1.47–3.11, P < 0.001), N stage (HR = 2.27, 95% CI 1.66–3.11, P < 0.001), AJCC stage (HR = 2.22, 95% CI 1.53–3.22, P < 0.001), LVI (HR = 2.25, 95% CI 1.55–3.29, P < 0.001), and risk score (HR = 2.72, 95% CI 1.61–4.60, P < 0.001) were associated with survival. Subsequent multivariate Cox analysis showed that age (HR = 1.87, 95% CI 1.21–2.89, P = 0.005), LVI (HR = 1.82, 95% CI 1.13–2.94, P = 0.014), and risk score (HR = 2.16, 95% CI: 1.11–4.20, P = 0.024) were independent prognostic indicators (Fig. 2d).

**Fig. 3:**
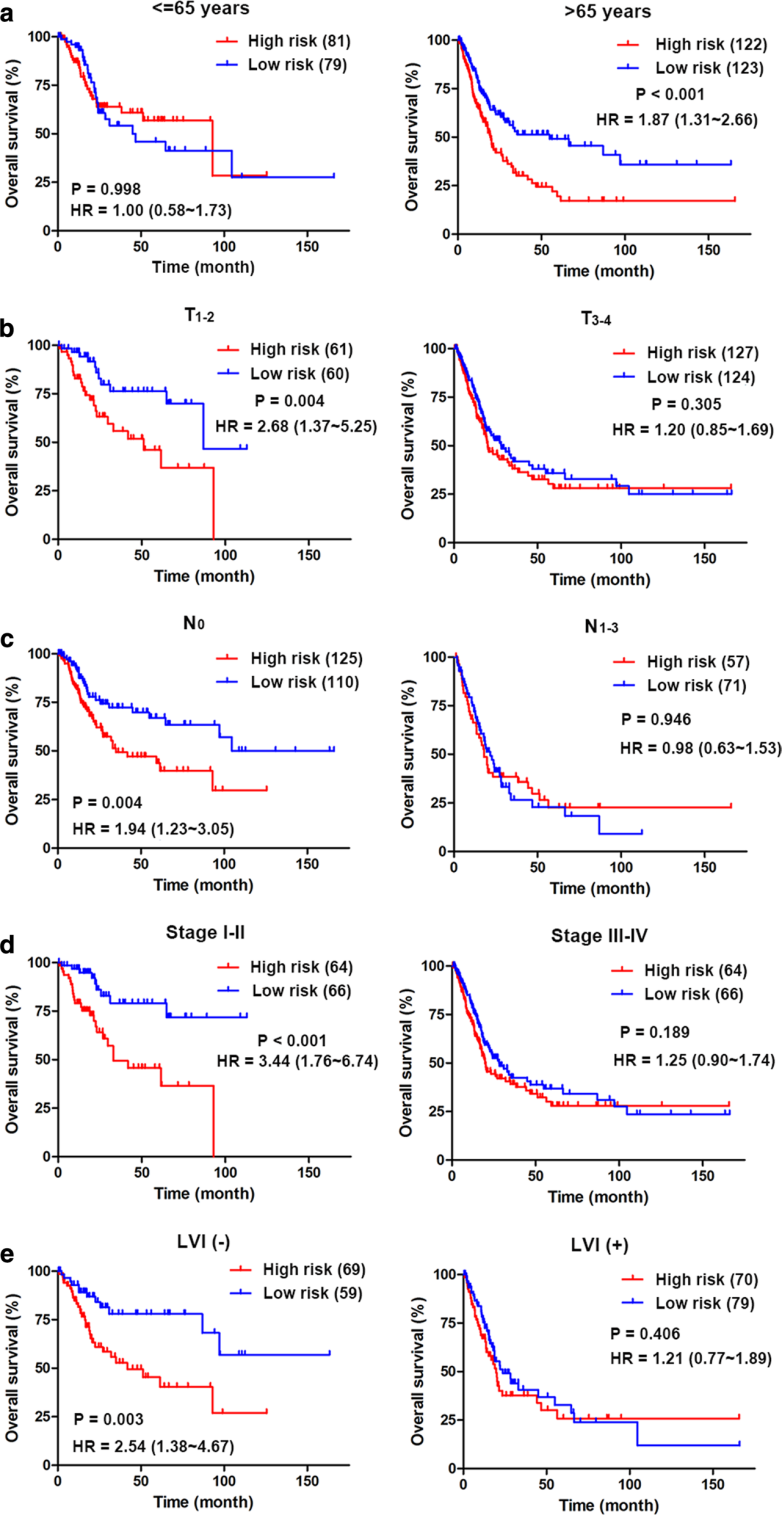
Stratification analysis of various clinicopathological factors by Kaplan–Meier curves for the patients with bladder cancer in the TCGA dataset. Kaplan–Meier curves of OS in different subgroups stratified by (**a**) age, (**b**) T stage, (**c**) N stage (**d**) AJCC stage and (**e**) LVI status

We next stratified the patients into different subgroups with median risk core according to age (≤ 65 versus > 65 years), T stage (T_1-2_ versus T_3-4_), N stage (N_0_ versus N_1-3_), AJCC stage (stage I–II versus stage III–IV) and LVI status [LVI(−) versus LVI(+)], and divided them into high-risk and low-risk groups based on the median risk score. Interestingly, high-risk scores suggested a poor prognosis in the elderly subgroup (HR = 1.87; 95% CI 1.36–2.66, P < 0.001; Fig. 3a), T_1-2_ subgroup (HR = 2.68; 95% CI 1.37–5.25, P = 0.004; Fig. 3b), N_0_ subgroup (HR = 1.94; 95% CI 1.23–3.05, P = 0.004; Fig. 3c), stage I–II subgroup (HR = 3.44; 95% CI 1.76–6.74, P < 0.001; Fig. 3d) and LVI (–) subgroup (HR = 2.54; 95% CI 1.38 ~ 4.67, P = 0.003; Fig. 3e); however, high-risk scores did not suggest a poor prognosis in the young, T_3-4_, N_1-3_, and stage III-IV and LVI (+) subgroups, suggesting that the 4-mRNA signature has a better prognostic value for patients with bladder cancer with low malignancy.

### Performance of the 4‑mRNA signature in the validation datasets

**Fig. 4:**
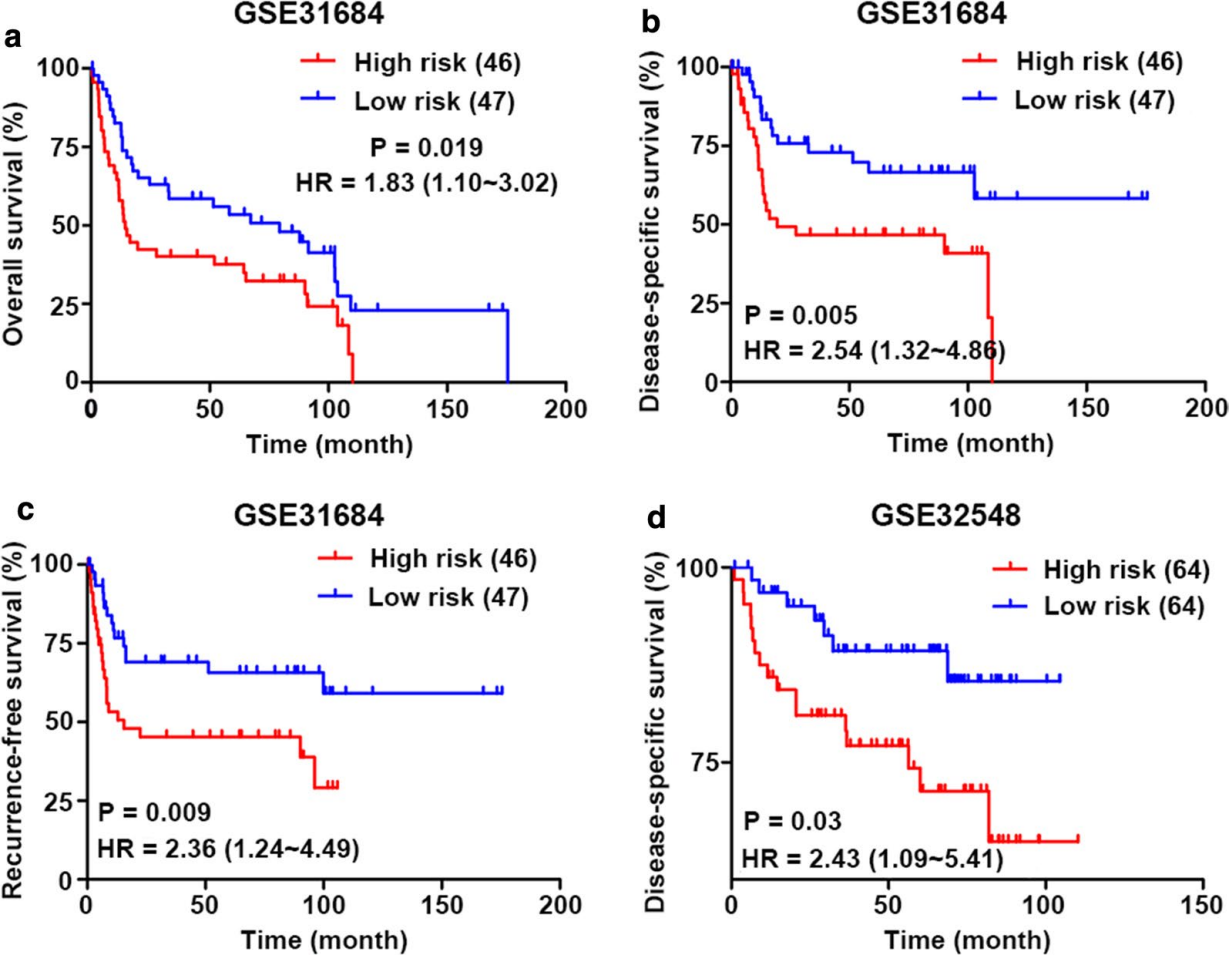
Validation of the 4‑mRNA signature for prognosis in two independent GEO datasets. Kaplan–Meier curves of (**a**) OS, (**b**) DSS and (**c**) RFS in the GSE31684 cohort. **d** Kaplan–Meier curves of DSS in the GSE32548 cohort

To further test the prediction value of the 4‑mRNA signature in different cohorts, GSE31684 and GSE32548 datasets from the GEO database were employed as external validation datasets. Among them, GSE31684 was used for OS, DSS and RFS validation, and GSE32548 was used for DSS validation. Patients in the validation cohorts were divided into a high‑risk group and a low‑risk group based on the median. Consistent with the performance in the TCGA training dataset described before, we found significant differences in OS, DSS and RFS between patients in the high‑risk and low‑risk groups in the GSE31684 dataset [high-risk vs low risk: HR = 1.83, 95% CI 1.10–3.02, P = 0.019; HR = 2.54, 95% CI 1.32–4.86, P = 0.005; HR = 2.36, 95% CI 1.24–4.49, P = 0.009; respectively] (Fig. 4a–c). Similarly, patients with a high risk score had a shorter DSS than those with a low risk score in the GSE32548 dataset (HR = 2.43, 95% CI 1.09–5.41, P = 0.03; Fig. 4d).

### Establishment of a nomogram incorporating the glycolysis-related gene signature

**Fig. 5:**
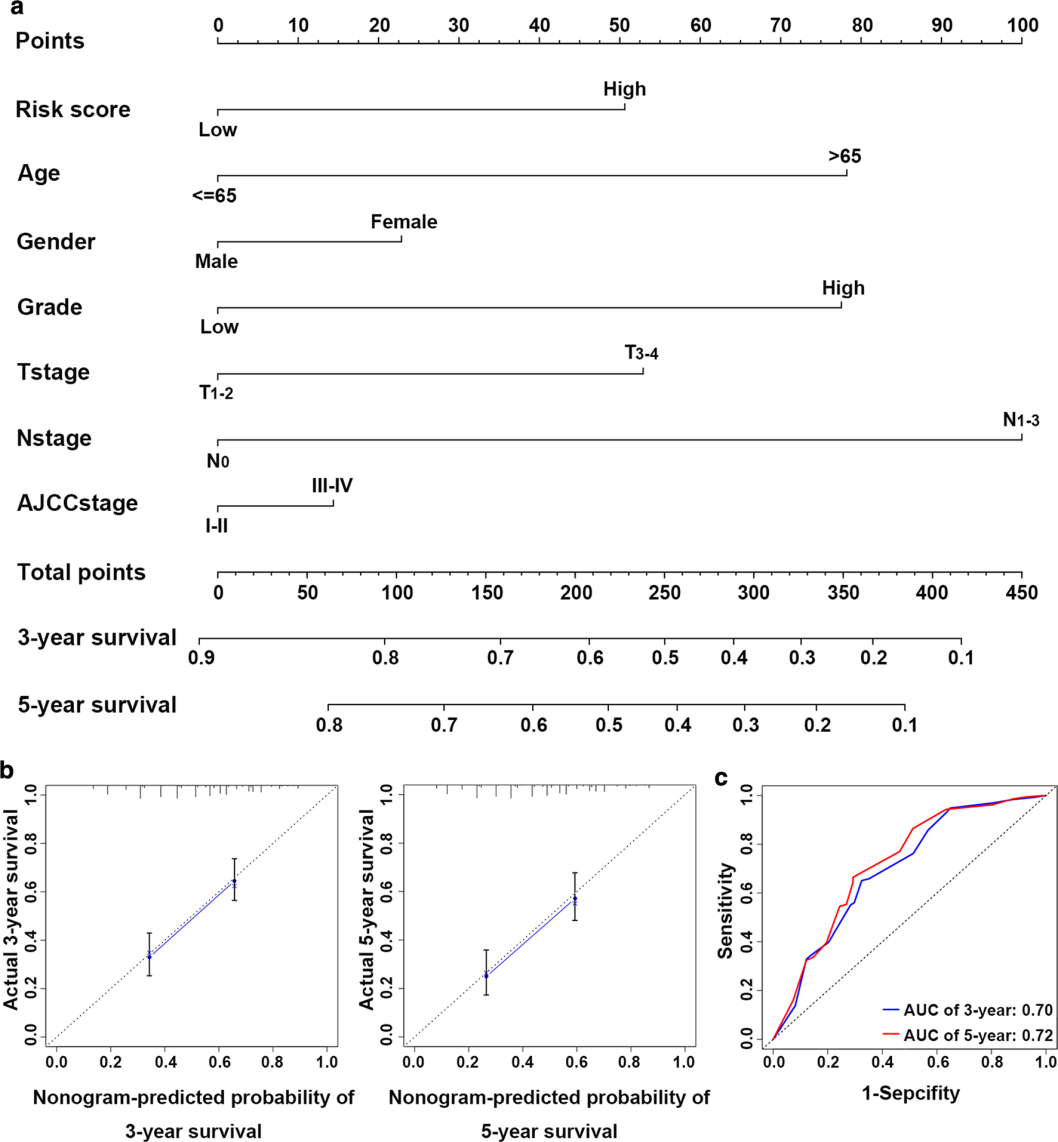
An established nomogram for predicting 3- and 5-year OS. **a** Nomogram incorporated with the 4-mRNA signature and clinical factors for prediction of the 3- and 5-year OS in patients with bladder cancer in the TCGA dataset. **b** Calibration curve of the nomogram for the prediction of 3- and 5-year OS. **c** Time‑dependent ROC curves based on the nomogram for 3‑ and 5‑year OS

To provide a clinically practical tool for clinicians to predict the probability of 3- and 5-year OS in patients with bladder cancer, we constructed a nomogram combining clinicopathological characteristics (age, gender, grade, T stage, N stage, and AJCC stage) and the 4-mRNA signature based risk score (Fig. 5a). In comparison to the ideal model, the calibration plots for 3‑year and 5‑year OS were good predictors (Fig. 5). Time‑dependent ROC curves showed that the AUC (area under curve) of the nomogram at 3‑ and 5‑year was 0.70 and 0.72, respectively (Fig. 5c).

### Analysis of network and biological processes associated with glycolysis-based genes

**Fig. 6:**
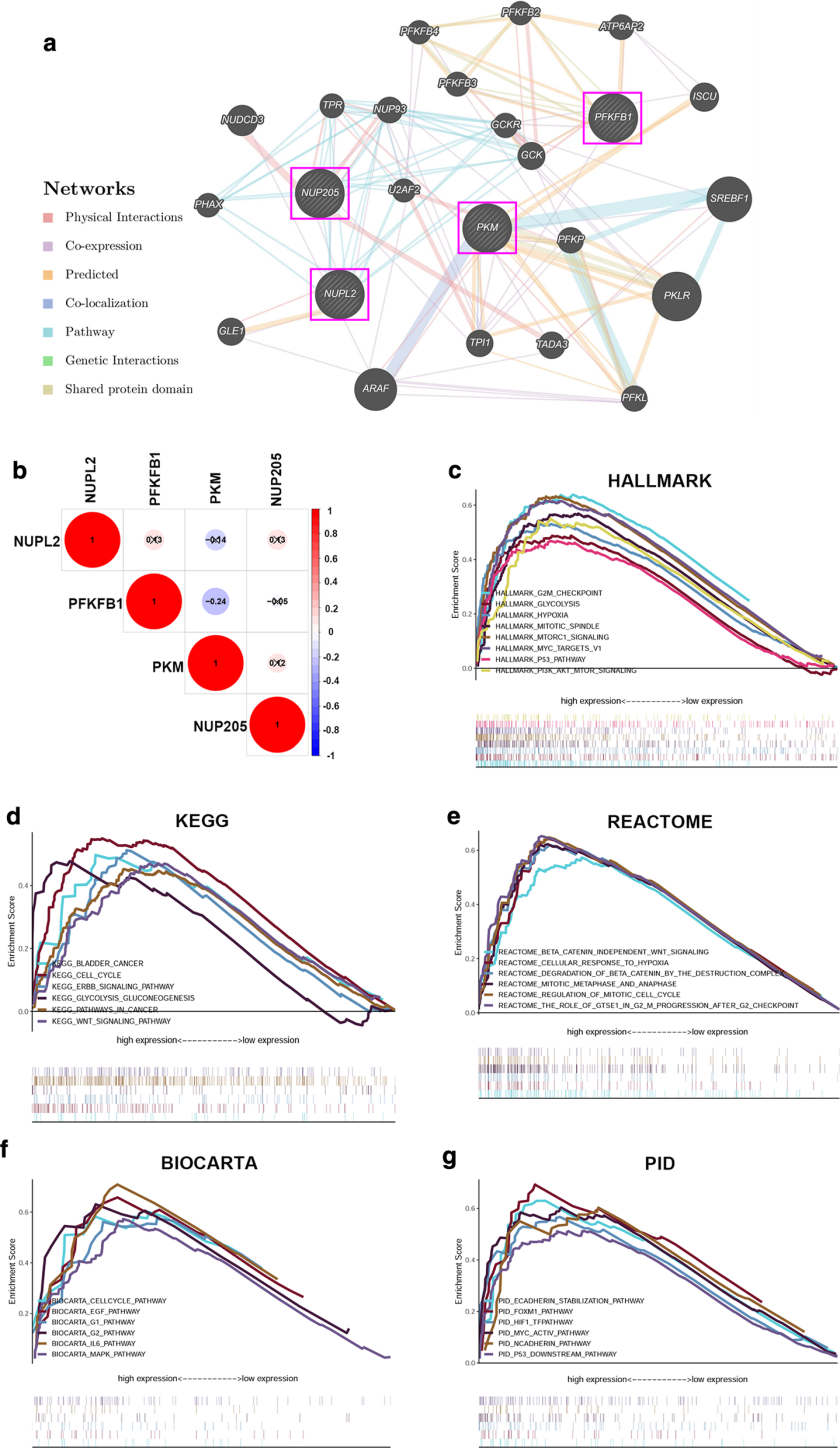
Analysis of the regulatory network and gene sets associated with the four glycolysis-related genes. **a** GeneMANIA constructed a protein‑protein interaction network involving these four genes. **b** Correlation between the four genes in the dataset from TCGA. Gene sets of (**c**) Hallmark, (**d**) KEGG, (**e**) Reactome, (**f**) BioCarta and (**g**) PID associated with 4-mRNA signature based risk score were performed by GSEA with nominal P‑value < 0.05, FDR < 0.1 and |NES| > 1.6

A gene regulatory network was constructed by GeneMANIA to determine the interactive relationships between the four glycolysis genes and other potential genes. The interaction network consisted of twenty-four genes, including four input target genes and twenty other genes that were spontaneously identified by GeneMANIA (Fig. 6a). The weights and connections of genes and the biological functions in the network are summarized in Additional file 4. We then analyzed the correlation of the four genes in bladder cancer and found that the absolute values of the correlation coefficient were all less than 0.3, suggesting that these genes are independent of each other (Fig. 6b).

GSEA was conducted to identify glycolysis-related biological processes and signaling pathways involved in carcinogenesis. The results indicated that ‘Hallmark’ gene sets involving cell cycle signaling, the PI3K/AKT/mTOR pathway, hypoxia and the p53 pathway that were related to biological processes of cancers were also enriched in the high‑risk group (Fig. 6c). In addition, several canonical pathways derived from BioCarta, KEGG, PID and Reactome gene sets, including the cell cycle pathway, the WNT/β-catenin signaling pathway, the p53 downstream pathway and the hypoxia response were highly enriched in the high‑risk phenotype (Fig. 6d–g).

### The glycolysis-related gene signature is enriched in the basal subtype and is positively correlated with the cell cycle

**Fig. 7:**
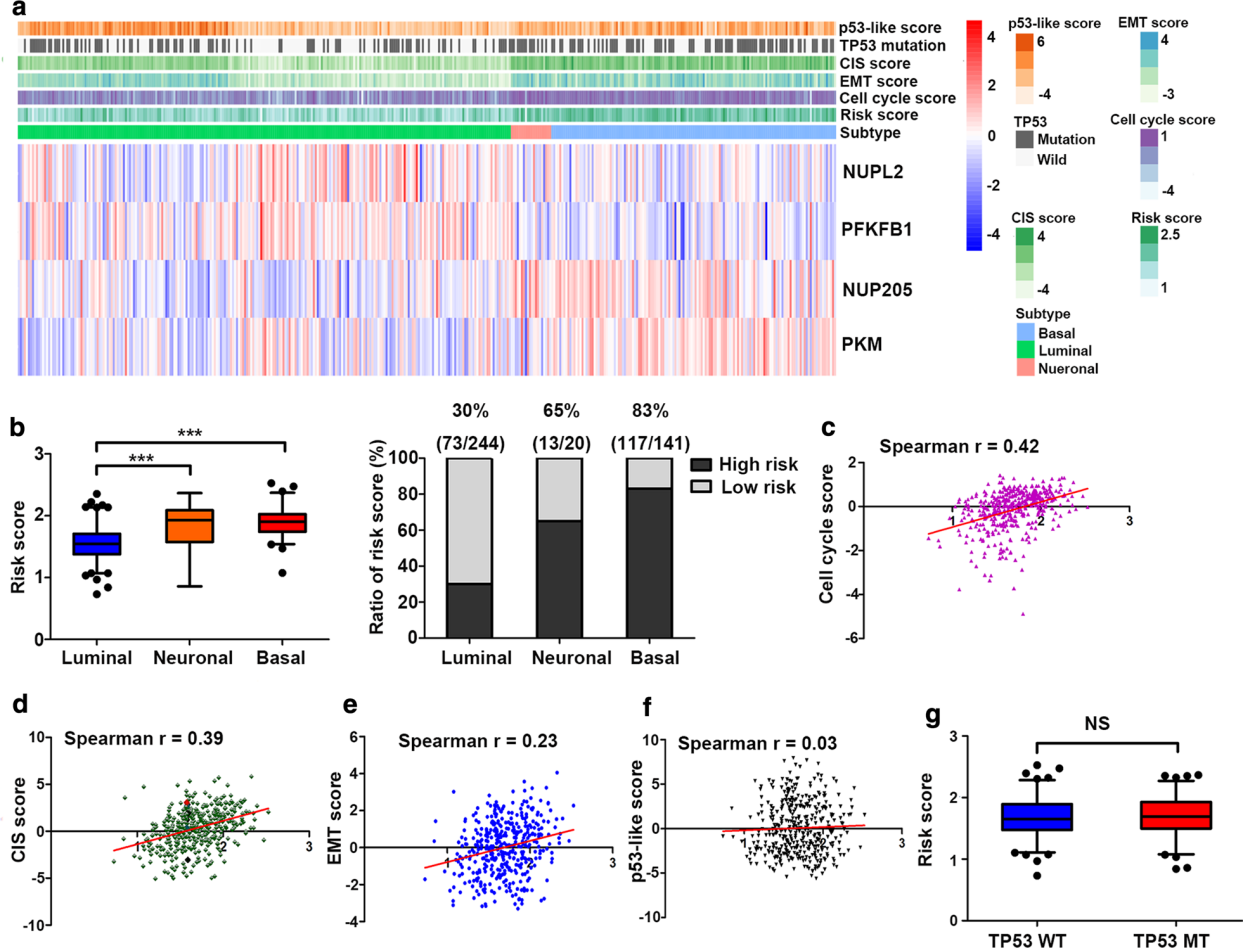
Relationship between glycolysis-related gene signature and molecular characterization of bladder cancer. **a** Heatmap showing the associations between the risk signature and the molecular characterization (p53-like signature, TP53 mutation, CIS signature, EMT signature, cell cycle signature, subtypes of bladder cancer) in the dataset from TCGA. **b** Distribution of the risk scores and the percentage of high-risk groups in different molecular subtypes of bladder cancer. **c** Correlation between cell cycle signature-based scores and risk score. **d** Correlation between CIS signature-based scores and risk score. **e** Correlation between EMT signature-based scores and risk score. **f** Correlation between p53-like signature-based scores and risk score. **g** Comparison of risk score in TP53-wildtype and TP53-mutant in the TCGA dataset. CIS, carcinoma in situ. WT, wild type. MT, mutant. NS, nonsignificant

The comprehensive molecular characterization of muscle-invasive bladder cancer based on multiplatform analysis of TCGA has largely improved our understanding of the heterogeneity of bladder cancer [10]. To investigate the relationship between the expression of glycolysis-related genes and the classification of bladder cancer as well as the molecular mechanisms and biological processes involved in cancer development, we explored the distributions of risk score in the following molecular subtypes of bladder cancer from TCGA cohort: p53-like signature, TP53 mutation, CIS signature, EMT signature, and cell cycle signature. The results showed that most patients with high risk scores were located in the basal and neuronal subtype groups of bladder cancer, and they had higher cell cycle, CIS and EMT signature scores (Fig. 7a, b). Patients with basal subtype had a poor overall survival than that with luminal subtype in Stage II  (Additional file 5). Relevant information is provided in Additional file 6. Analysis of correlation between the risk score and several signature scores indicated that glycolysis-related signature was closely related to CIS and cell cycle process (Fig. 7c, d), had a certain correlation with EMT (Fig. 7e), and had no correlation with p53-like signature (Fig. 7f). Additionally, there were no significant differences in risk scores between the TP53 wild type (WT) group and the TP53 mutant (MT) group (Fig. 7g).

### Expression levels of the four glycolysis genes in bladder cancer

**Fig. 8:**
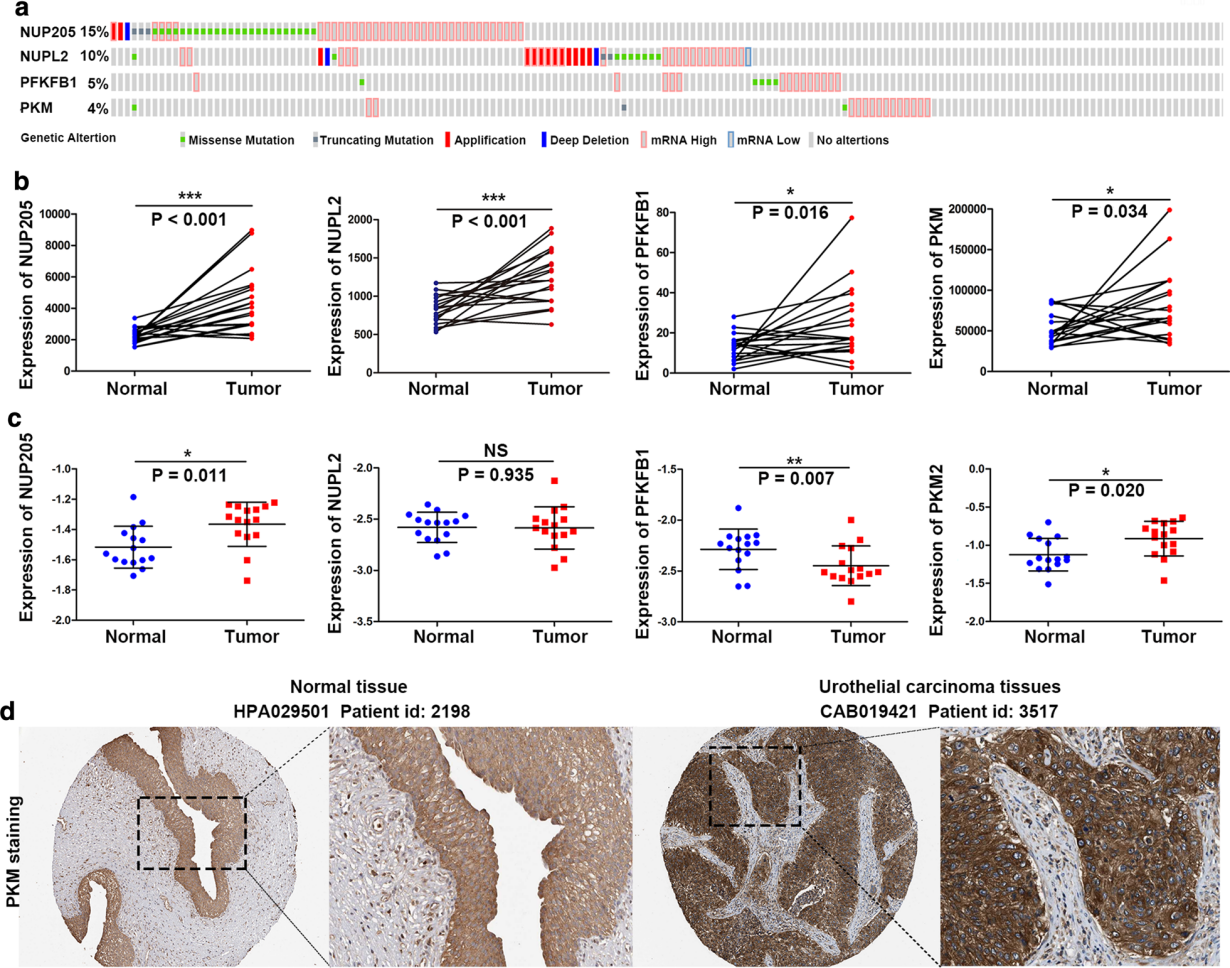
Identification of the four glycolysis-related genes. **a** The proportion of alteration for the four genes in 408 clinical samples of bladder cancer in the cBioPortal database. **b** Expression of the four genes in the bladder cancer samples (n = 19) and paired adjacent normal samples (n = 19) in the dataset from TCGA. **c** Expression of the four genes in bladder cancer tissues (n = 15) and normal tissues (n = 15), as detected by RT-qPCR. **d** IHC staining of PKM protein in normal urothelial tissues and bladder cancer tissues in the Human Protein Atlas database. *P < 0.05, **P < 0.01, and ***P < 0.001. NS: nonsignificant

Genetic alterations of the four target genes were analyzed via the cBioPortal database, which contains data on 408 bladder cancer cases from the TCGA database. The results showed that the queried genes were altered in 119 (29.2%) sequenced cases. As shown in Fig. 8a, NUP205 exhibited a mutation with a frequency of 15%, NUPL2 at 10%, PFKFB1 at 5% and PKM at 4%. The differential expression of the four genes in adjacent normal and bladder cancer tissues was also investigated. The four genes were all significantly upregulated in the cancer samples in the cohort from TCGA (Fig. 8b). However, using RT-qPCR for further validation, we found that only NUP205 and PKM were upregulated in cancer tissues, and the expression of PFKFB1 in cancer tissues was lower than that in adjacent normal tissues (Fig. 8c). Immunohistochemistry (IHC) staining images from the Human Protein Atlas database (https://www.proteinatlas.org/) showed that PKM intensity in urothelial carcinoma tissues was stronger than that in urothelial mucosa (Fig. 8d).

### Knockdown of PKM2 inhibits cell proliferation in bladder cancer

**Fig. 9:**
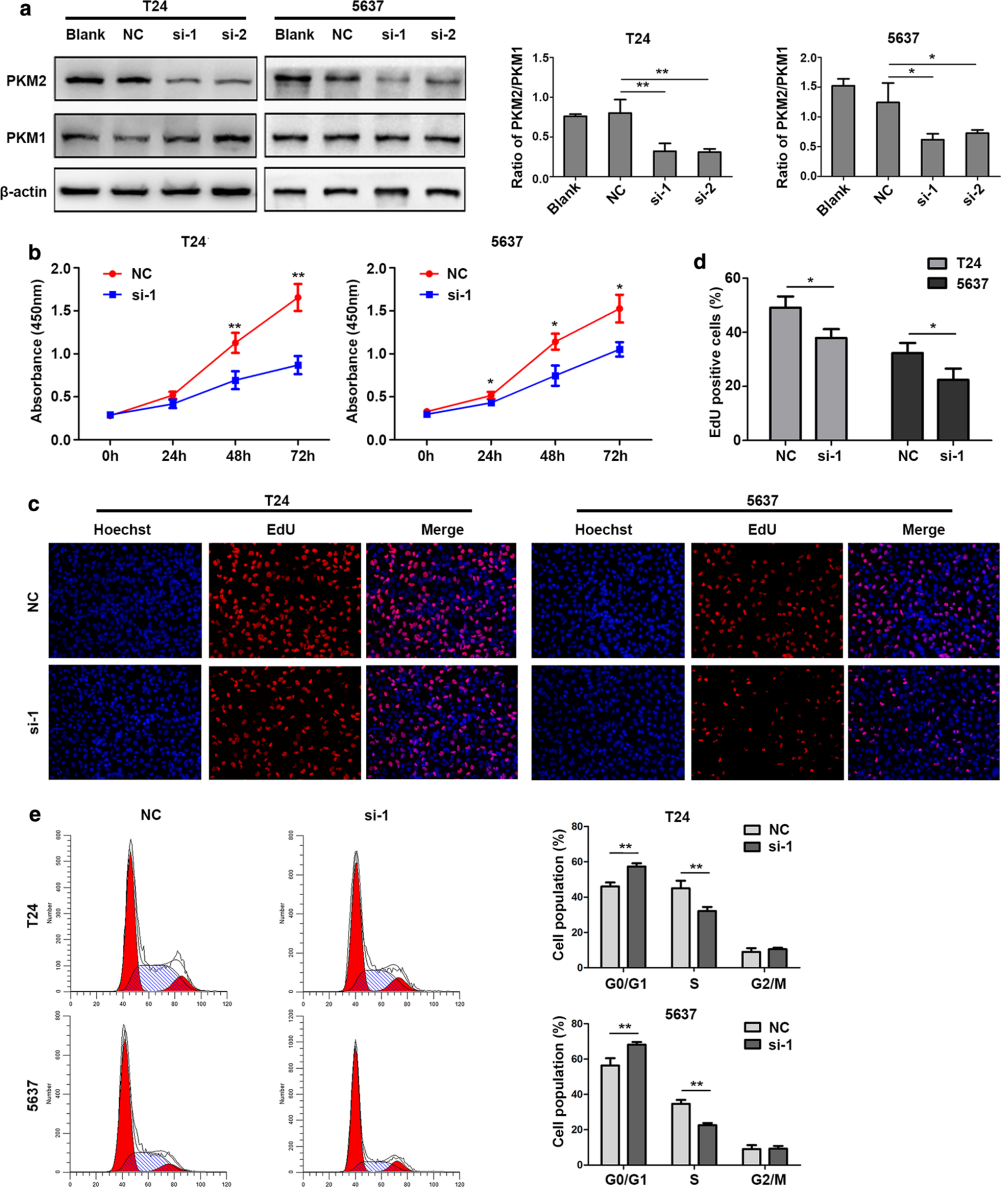
Effect of PKM2 on cell proliferation in bladder cancer cells. **a** PKM2 and PKM1 expression in T24 and 5637 cells transfected with siRNAs determined by Western blot. Data are presented as the mean ± SD, n = 3, **p < 0.01 and ***P < 0.001. **b** Cell growth was detected by CCK8 assay in T24 and 5637 cells after transfection with NC or PKM2 siRNA. Data are presented as the mean ± SD, n = 3, *p < 0.05 and **P < 0.01 vs NC. **c**, **d** Cell proliferation was detected using the EdU assay in T24 and 5637 cells (100× magnification). Data are presented as the mean ± SD, n = 3, *p < 0.05. **e** Flow cytometric analysis of the cell cycle distribution in T24 and 5637 cells post-transfection with NC or PKM2 siRNA. The percentages of cells in G0/G1, S and G2/M phase are shown in the bar graph. Data are presented as the mean ± SD, n = 3, **p < 0.01. NC, negative control. si-1, PKM2 siRNA-1. si-2, PKM2 siRNA-2

To study the role of PKM2 in bladder cancer cells, T24 and 5637 cells were transfected with two separate siRNAs targeting PKM2 and with a negative control (NC) siRNA. The expression of PKM2 and PKM1 was determined by Western blot. The results demonstrated that, compared with the blank and NC groups, the expression of PKM2 protein was significantly decreased in T24 and 5637 cells after transfection with siRNA-1 and siRNA-2, and there was no effect on the PKM1 expression level (Fig. 9a), suggesting a great specificity of siRNA targeting PKM2, herein we chose siRNA-1 for subsequent study.

The effect of PKM2 on cell proliferation was evaluated using CCK-8, EdU and cell cycle assays. The CCK-8 assay demonstrated that cell proliferation was inhibited in T24- and 5637-siPKM2 cells compared to NC cells (Fig. 9b). The EdU assay showed that downregulation of PKM2 suppressed proliferation of T24 and 5637 cells, with a decreased proportion of cells in the DNA synthesis phase (Fig. 9c, d). Furthermore, the results of flow cytometry for cell cycle distribution indicated that the percentage of cells in the G0/G1-phase was greater in the siPKM2 group than in the NC group for T24 and 5637 cells (Fig. 9e). These results suggest that PKM2 is involved in the cell growth of bladder cancer, which may be mediated by regulation of the cell cycle.

## Discussion

Since altered glucose metabolism has been regarded as a hallmark of cancers, energy metabolism has attracted extensive attention in oncology research in recent years. Glycolytic intermediates provide nutrients necessary for the viability and proliferation of cancer cells, and excessive accumulation of lactate creates an acidic microenvironment, driving tumor invasion and metastasis and conferring resistance to radiation therapy [15, 16]. Thus, the Warburg effect is a favorable pathway used by tumor cells to harness cellular stress to enable their thriving. Increased aerobic glycolysis has been shown to contribute to tumor aggressiveness in bladder cancer cells [17]. Loss of the glycogen debranching enzyme causes rapid proliferation of bladder cancer cells and has prognostic value for bladder cancer patients [18]. Therefore, glycolysis status may become an emerging hallmark of tumor malignancy and a potential indicator used for the prognosis of patients with bladder cancer. Our results showed that glycolysis analysis using the 4-mRNA signature is an effective way to independently generate prognoses for bladder cancer patients. Moreover, we found that patients with high glycolysis-related risk scores mainly had basal and neuronal subtypes of bladder cancer with poor prognosis. Notably, the risk score based on the 4-mRNA signature positively correlated with the cell cycle and CIS signatures, which was further validated by in vitro experiments, where knocking down PKM2 increased cell cycle arrest at the G0/G1 phase in bladder cancer cells.

Recent studies have shown that traditional clinicopathological parameters are insufficient for accurately predicting the prognosis of cancer patients. Comprehensive genomic studies based on high throughput RNA sequences and microarray profiles have been utilized to develop molecular signatures for predicting the outcomes of various clinical diseases [19]. Glycolysis-related gene signatures have an excellent performance in predicting clinical outcomes for multiple types of solid tumors. For instance, a set of nine genes derived from the gene set of Hallmark glycolysis were significantly associated with overall survival and metastasis in patients with lung adenocarcinoma, and those with higher risk scores had a poorer prognosis [20]. Similarly, another concurrent study showed that the glycolysis-related signature composed of four genes was closely related to the prognosis of patients with lung adenocarcinoma [21]. A three-gene glycolytic signature (MET, B3GNT3 and SPAG4) can act as an independent factor for generating a prognosis for patients with pancreatic ductal adenocarcinoma [22]. A nine‑gene signature associated with cellular glycolysis was a potent indicator for the prediction of overall survival in patients with endometrial cancer [23]. The glycolysis score represented by expression of ten glycolytic genes predicted unfavorable clinical outcome for patients with glioblastoma and was closely related to mesenchymal subtype and mutation of IDH1 in glioblastoma [24]. Consistent with those studies, we found that a glycolysis signature composed of four glycolytic genes (NUP205, NUPL2, PFKFB1 and PKM) can predict the survival of patients with bladder cancer accurately in the TCGA cohort as well as in two independent GEO cohorts, suggesting its favorable performance for prediction. In addition, a nomogram incorporated with glycolysis risk score and clinical factors had better prognostic value and higher potential for clinical utility than a single parameter.

Pyruvate kinase (PK) is a key enzyme that catalyzes the final step of glycolysis by transferring phosphoenolpyruvate to pyruvate and converting ADP to ATP. There are two alternative splice isoforms of PK, PKM1 and PKM2. PKM1 is expressed in normal differentiated tissues, such as skeletal muscle, heart and brain. PKM2 is initially expressed in proliferating cells and during embryonic development; it increases glycolysis even in an aerobic environment [25, 26]. The PKM subtype can be switched from PKM1 to PKM2 in rapidly proliferating tumor cells, which is involved in the loss of pyruvate kinase activity [27]. PKM2 was found to be the prominent isoform in bladder cancer samples, accounting for approximately 60% of PKM in the bladder [28]. Another study also revealed that PKM2, but not the spliced variant PKM1, was upregulated in low-grade and, more prominently, high-grade bladder cancer. Specific inhibition of PKM2 decreased cell proliferation by increasing apoptosis, autophagy and the unfolded protein response in bladder cancer cells [29]. Consistently, our study found that the mRNA level of PKM was upregulated in bladder cancer tissues compared with normal tissues. Knockdown of PKM2 by siRNA suppressed cell proliferation in T24 and 5637 cells, suggesting that PKM2 may have potential value in the diagnosis and treatment of bladder cancer.

There is a close interconnection between glucose metabolism and the cell cycle in cancer. A variety of glycolytic enzymes perform nonmetabolic functions to sustain tumor proliferation, invasiveness, and metastatic ability [30, 31]. Several of them are periodically shifted into the nucleus, linking metabolism to cell cycle progression. Like cyclins, their activities are kept in dynamic equilibrium via degradation that is mediated by similar ubiquitin–proteasome system [32]. Periodic activation of cyclins-CDKs and metabolic enzymes drives cell cycle progression. Glycolytic enzymes, such as HK2, the inactive form of PKM2, and PFKFB3, are mainly conducive to protein synthesis in the G1 phase. GAPDH and the active form of PKM2 are activated during the G2 phase [33]. As a protein kinase, PKM2 phosphorylates STAT3 and histone H3, increasing the transcription levels of c-Myc, STAT3, and HIF-1 [30, 34, 35]. PKM2 that is activated by some proliferative signaling pathways, such as AKT, EGFR, and NFκB, sustains cell cycle progression and promotes transcription of glycolytic enzymes and glutamine synthase 1 [36]. In this study, we found that the glycolysis-related risk score was closely associated with the cell cycle progression score derived from 31 cell cycle proliferation genes [37]. Furthermore, downregulation of PKM2 by siRNA treatment caused cell cycle arrest at G0/G1 phase in bladder cancer cells, which is supported by previous studies showing that dimeric PKM2 enhances cellular biosynthesis and expression of cyclin D1 by upregulating the expression of c-Myc and promoting nuclear translocation of β-catenin [38], and PKM2 directly phosphorylates histone H3 at T11, resulting in H3-K9 acetylation and transcription of genes, including CCND1 [39]. These findings demonstrated that glycolytic enzymes play an important role in regulation of the cell cycle.

The 4-mRNA signature based on glycolysis shows an effective model for predicting the prognosis of patients with bladder cancer. However, our research also had certain limitations. The study may cause a selection bias due to the retrospective feature. The sample size was insufficient in the validation datasets. The predictive model, therefore, need to be further validated in large prospective clinical trials. Moreover, the specific mechanisms of PKM2 to modulate bladder cancer cells at G0/G1 phase need further study. In summary, our results demonstrated that the glycolysis-related 4-mRNA signature shows potential roles in the prediction of clinical outcome and in enabling personalized therapy in patients with bladder cancer. In addition, PKM2 is involved in the regulation of cell proliferation and cell cycle progression in bladder cancer cells.

## Conclusion

We constructed a glycolysis based 4-mRNA signature to predict prognosis for patients with bladder cancer. The 4-gene signature was an independent prognostic factor for overall survival. Its performance on prognostic prediction was further validated in two independent cohorts from GEO database. GSEA was conducted to explore the 4-mRNA related canonical pathways and biological processes. In addition, the signature-based risk score was significantly associated with the cell cycle process. Loss of PKM2 by RNAi led to cell cycle arrest at G0/G1 phase in bladder cancer cells. These results suggest that 4‑mRNA signature may not only act as a novel tool for predicting the clinical outcome of patients with bladder cancer, but also provides insight into the mechanisms of cellular glycolysis in carcinogenesis.

## Supplementary information

**Additional file 1.** Risk scores, expression signature scores, TP53 mutation and mRNA subtypes of bladder cancer in the TCGA cohort.

**Additional file 2.** Ethical approval notice.

**Additional file 3.** Clinicopathological characteristics of fifteen patients.

**Additional file 4.** Modes of interaction in PPI network.

**Additional file 5.** Kaplan-Meier curves for the bladder cancer patients in Stage II with basal_squamous and luminal subtypes in the TCGA cohort.

**Additional file 6.** Basal_squamous and luminal subtypes in Stage II patients with bladder cancer in the TCGA cohort.

**Additional file 7.** Certificate of language modification.

## Data Availability

The information of this study here is obtained from the TCGA (https://portal.gdc.cancer.gov/), GEO (https://www.ncbi.nlm.nih.gov/geo/), Molecular Signatures Database (https://www.gsea-msigdb.org/gsea/msigdb/genesets.jsp), GeneMANIA (http://www.genemania.org/), cBioportal (http://www.cbioportal.org/), and Human Protein Atlas (https://www.proteinatlas.org/).

## Abbreviations

TCGA: The Cancer Genome Atlas
GEO: Gene Expression Omnibus
GSEA: Gene set enrichment analysis
ROC: Receiver operating characteristic
RT-qPCR: Reverse transcriptase quantitative polymerase chain reaction
NMIBC: Non-muscle‐invasive bladder cancer
MIBC: Muscle‐invasive bladder cancer
OS: Overall survival
DSS: Disease-specific survival
RFS: Recurrence-free survival
EMT: Epithelial–mesenchymal transition
CIS: Carcinoma-in situ
FDR: False discovery rate
NES: Normalized enrichment score
ATCC: American Type Culture Collection
CCK8: Cell Counting Kit-8
HR: Hazard ratio
LVI: Lymphovascular invasion
AJCC: American Joint Committee on Cancer
CI: Confidence interval
WT: Wild type
MT: Mutant
NC: Negative control
