# Supplementary figures and images for: A glycolysis-based 4-mRNA signature correlates with the prognosis and cell cycle process in patients with bladder cancer
Source: Cancer Cell Int. 2020 May 20;20:177. doi: 10.1186/s12935-020-01255-2 (PMC7238531; doi:10.1186/s12935-020-01255-2)

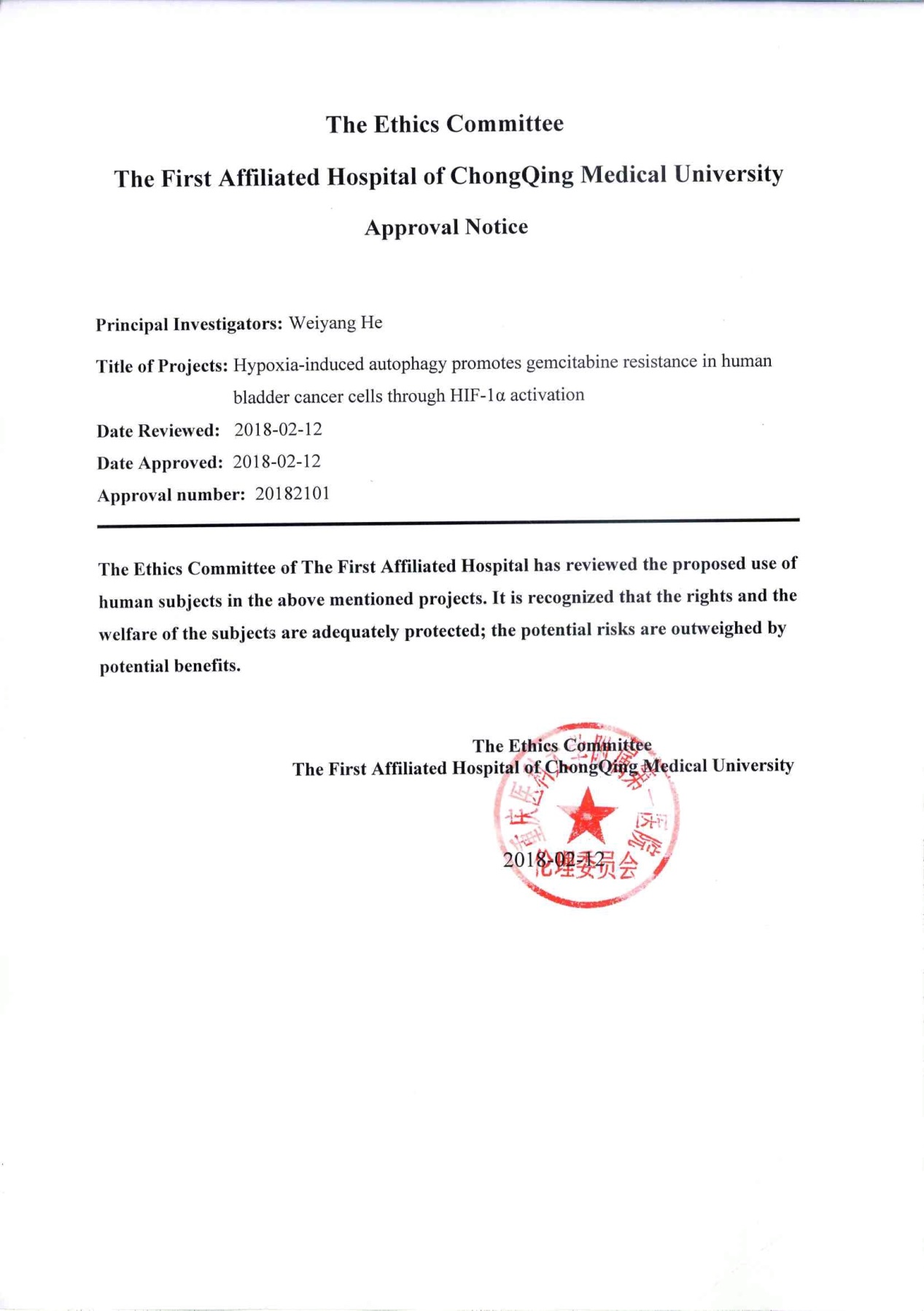

Supplement: Supplementary file 2 — Additional file 2. Ethical approval notice. [file 12935_2020_1255_MOESM2_ESM.docx]

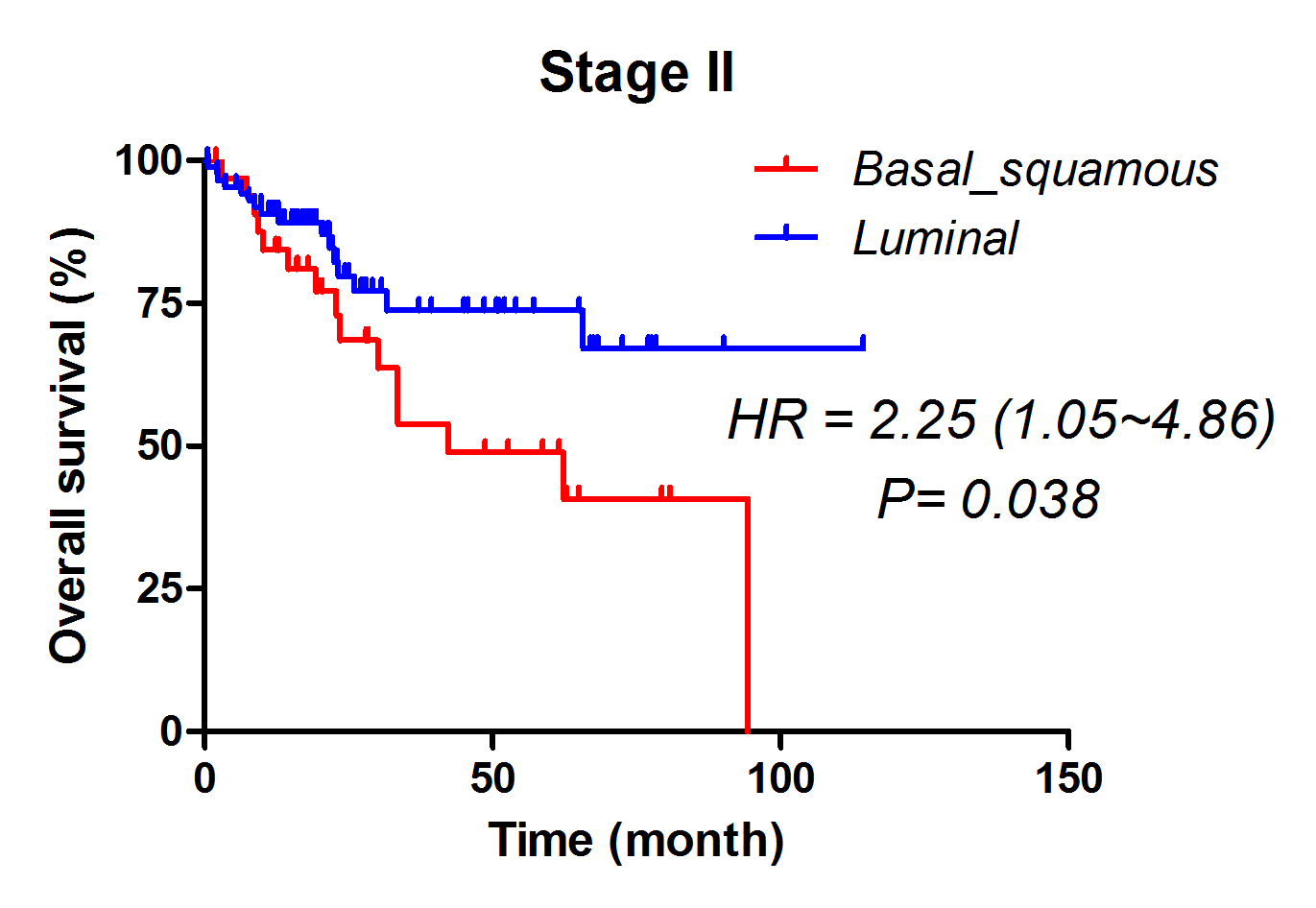

Supplement: Supplementary file 5 — Additional file 5. Kaplan-Meier curves for the bladder cancer patients in Stage II with basal_squamous and luminal subtypes in the TCGA cohort. [file 12935_2020_1255_MOESM5_ESM.tif]
