# Supplementary material for: A glycolysis-based 4-mRNA signature correlates with the prognosis and cell cycle process in patients with bladder cancer
Source: Cancer Cell Int. 2020 May 20;20:177. doi: 10.1186/s12935-020-01255-2 (PMC7238531; doi:10.1186/s12935-020-01255-2)
Supplement: Supplementary file 3 — Additional file 3. Clinicopathological characteristics of fifteen patients. [file 12935_2020_1255_MOESM3_ESM.docx]

Table. Clinicopathological parameters of patients enrolled in the cohort.

| Characteristics | Value |
| --- | --- |
| Number of patients | 15 |
| Age (mean ± SD) | 65.53 ± 8.97 |
| Gender (Male/Female) | 2/13 |
| Pathology grade (Low/High) | 2/13 |
| Tumor size (≤ 3cm/> 3cm) | 6/9 |
| Subtype (Papillary/Non-papillary) | 11/4 |
| T stage (Ta-T_1_/T_2_-T_4_) | 1/14 |
| Lymph node metastasis (No/Yes) | 15/0 |
| TNM stage (I/II/III/IV) | 1/10/2/2 |

SD, standard deviation; TNM, tumor-node-metastasis
