# Supplementary material for: A glycolysis-based 4-mRNA signature correlates with the prognosis and cell cycle process in patients with bladder cancer
Source: Cancer Cell Int. 2020 May 20;20:177. doi: 10.1186/s12935-020-01255-2 (PMC7238531; doi:10.1186/s12935-020-01255-2)
Supplement: Supplementary file 7 — Additional file 7. Certificate of language modification. [file 12935_2020_1255_MOESM7_ESM.pdf]

Editing Certificate

This document certifies that the manuscript

A glycolysis-based 4-mRNA signature correlates with the prognosis and cell cycle process in patients with bladder cancer

prepared by the authors

Chen Zhang, Xin Gou, Weiyang He, Huaan Yang, Hubin Yin

was edited for proper English language, grammar, punctuation, spelling, and overall style by one or more of the highly qualified native English speaking editors at AJE.

This certificate was issued on **March 22, 2020** and may be verified on the AJE website using the verification code **C0F0-7622-59DD-7130-F489**.

Neither the research content nor the authors' intentions were altered in any way during the editing process. Documents receiving this certification should be English-ready for publication; however, the author has the ability to accept or reject our suggestions and changes. To verify the final AJE edited version, please visit our verification page at aje.com/certificate. If you have any questions or concerns about this edited document, please contact AJE at support@aje.com.

AJE provides a range of editing, translation, and manuscript services for researchers and publishers around the world. For more information about our company, services, and partner discounts, please visit aje.com.
